# Molecular Networking‐Driven Chemical Profiling, Characterization, and Antibacterial Effects of Cuo Nanoparticles Synthesized from Citrus Unshiu Peel Extract

**DOI:** 10.1002/open.202500374

**Published:** 2025-09-19

**Authors:** Livhuwani Mafhala, Shohreh Azizi, Garland More, Ilunga Kamika

**Affiliations:** ^1^ UNESCO‐UNISA Africa Chair in Nanoscience and Nanotechnology College of Graduates Studies University of South Africa Muckleneuk Ridge Pretoria 392 South Africa; ^2^ CAES Laboratories College of Agriculture and Environmental Sciences University of South Africa Johannesburg Gauteng South Africa; ^3^ Institute of Nanotechnology and Water Sustainability School of Science College of Science, Engineering, and Technology, Florida Campus University of South Africa Florida, Roodepoort Johannesburg Gauteng 1709 South Africa

**Keywords:** antimicrobial activities, *Citrus unshiu*, copper oxide nanoparticles, green syntheses, liquid chromatography‐–mass spectrometry, molecular networkings

## Abstract

Molecular networking has emerged as a new in silico tool for analyzing liquid chromatography–mass spectrometry (LC–MS) data for better annotation and elucidation of novel compounds and different pathways. Green synthesized nanoparticles (NPs) have gained considerable attention as a result of their effectiveness in possessing good antimicrobial activity. Their eco‐friendly nature and cost‐effective synthesis have positioned them as sustainable nanomaterials in various fields. However, not much is known about the mechanism underlying the green synthesis of NPs. Therefore, herein, the copper oxide NPs (CuO NPs) are fabricated, and ultrahigh performance liquid chromatography‐quadrupole time of flight mass spectrometry based molecular networking is utilized to understand the phytochemical relationship between the crude extract and the NPs, outlining metabolites that might be involved in reduction. Moreover, CuO NPs synthesized from *Citrus unshiu* fruit peels are tested for their antimicrobial and cytotoxic activities. Various characterization methods, such as X‐ray diffraction, UV–vis spectroscopy, scanning electron microscopy‐energy dispersive X‐ray analysis, Fourier transform infrared, dynamic light scattering, and transmission electron microscopy, are employed to provide comprehensive insights into the atomic and structural characteristics of NPs. Molecular network reveals the presence of different metabolites such as isosakuranetin‐7‐O‐rutinoside, hesperidin, skullcapflavone II, homoorientin, eupatorin‐5‐methylether, scoparin, and vitexin, which are recognized as antimicrobial and reducing agents. Additionally, the synthesized CuO NPs show exceptional antibacterial efficacy with a low minimum inhibitory concentration on *Staphylococcus aureus, Bacillus cereus, Escherichia coli,* and *Salmonella typhimurium*, highlighting the potential of using LC–MS to explain the antimicrobial properties and green synthesis pathway.

## Graphical abstract

Copper oxide nanoparticles (CuO NPs) were synthesized from Citrus unshiu peel extract and characterized by X‐ray diffraction (XRD), ultraviolet–visible spectroscopy (UV–Vis), Fourier‐transform infrared spectroscopy (FTIR), scanning electron microscopy (SEM), energy‐dispersive X‐ray spectroscopy (EDX), dynamic light scattering (DLS), and transmission electron microscopy (TEM).Ultra‐high performance liquid chromatography–quadrupole time‐of‐flight mass spectrometry (UHPLC–qTOF–MS) with Global Natural Products Social Molecular Networking (GNPS) showed flavones as reducing agents.

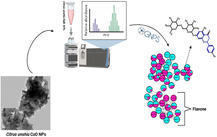

## 1 Introduction

The growing resistance of bacteria to traditional antibiotics has made it essential to explore novel antimicrobial agents, making the synthesis of nanoparticles (NPs) a promising approach in medical research.^[^ ^1^ ^]^ Copper oxide NPss (CuO NPs) have attracted remarkable interest as a result of their potent antibacterial efficacy and potential uses in pharmaceuticals. CuO NPs have the capacity to change various applications in areas such as drug delivery, antimicrobials, pest management, and parasitology.^[^ ^2^, ^3^ ^,^^]^

Among several NPs synthesis methods, the biosynthesis mediated by plants offers several advantages over both physical and chemical methods, including being cost‐effective, a single‐step process, and not requiring energy, high pressure, or temperature, nor the utilization of highly hazardous chemicals.^[^ 4, 5, 6 ^]^ Utilizing the products derived from plants has emerged as a successful and environmentally friendly technique for NPs preparation,^[^ ^7^ ^]^ owing to their phytochemicals that help reduce metal ions to NPs in a single step, including polyphenols, flavonoids, triterpenes, tannins, proteins, and steroids. This approach allows for exact regulation of NPs’ size distribution and crystallinity.^[^ ^8^ ^]^ *Citrus unshiu* peel extract is rich in diverse phytochemicals, provides an ideal matrix for the biosynthesis of CuO NPs while enhancing their therapeutic efficacy.^[^ ^9^ ^]^

Phytochemicals, the bioactive compounds found in plant extracts, play crucial roles in various biological functions, including antimicrobial activities, which can reduce inflammation.^[^ ^10^ ^,^ ^11^ ^]^ Compounds such as flavonoids, phenolic acids, and essential oils not only contribute to the reduction of microbial growth but also support overall health by mitigating inflammation.^[^ ^10^ ^,^ ^12^ ^]^ Notably, these phytochemicals also serve as capping, reducing, and stabilizing agents during the preparation of NPs, facilitating the formation of CuO NPs while ensuring their functionality and stability.^[^ ^13^ ^,^ ^14^ ^]^ Understanding the diverse roles of these phytochemicals in the context of nanoparticle synthesis is essential for harnessing their full potential.

The application of sophisticated analytical methods, including mass spectrometry and liquid chromatography (LC–MS), alongside the principle of molecular networking, allows for the comprehensive profiling and characterization of plant extracts, phytochemicals, and green‐synthesized NPs. Molecular networking facilitates the visualization of complex interactions among compounds, revealing their functional relationships and enabling the identification of key bioactive components present in NPs that aid in antimicrobial activity.^[^ ^15^ ^,^ ^16^ ^]^

In this study, CuO NPs were prepared using *C. unshiu* fruit peel extract, a bioactive waste for performing reduction and stabilization of NPs. The utilization of *C. unshiu* peels offers a sustainable and scalable approach, leveraging an abundant agro‐industrial waste material. The availability of flavonoids and polyphenols on the peels makes them capable of reducing copper ions and stabilizing the resulting NPs without the need for toxic chemicals. Given its widespread cultivation in many parts of the world and the large volume of peel waste generated during juice production and consumption, this biomass is readily available and underutilized.

Phytochemical composition of the extract and the NPs was assessed using quadrupole time‐of‐flight mass spectrometry for ultrahigh‐performance liquid chromatography (UHPLC‐QTOF‐MS). This study explored the synthesis of CuO NPs from *C. unshiu* peel extract, characterized their antibacterial effects, and elucidated the phytochemical composition through molecular networking and LC–MS analysis. Through this research, we seek to contribute valuable insights into the advancement of eco‐friendly antimicrobial substances that are capable of addressing the pressing issue of antibiotic resistance. Furthermore, we seek to test the ability of molecular networking in identifying potential phytochemicals present in NPs that aid in the antimicrobial effect of the green‐synthesized NPs and further unleash the green synthesis pathway of nanoparticle fabrication. To confirm the fabrication of the NPs prior to running the samples on LCMS, the as‐prepared NPs were characterized using different characterization techniques. Moreover, the antibacterial activity and safety of the fabricated CuO were studied with two gram‐negative and two gram‐positive strains.^[^ ^17^ ^]^ Finally, research on the cytotoxicity of CuO was conducted employing both healthy and cancerous cells to determine viability under different application conditions.

## 2 Results and Discussion

### 2.1 Antimicrobial and Cytotoxicity Activity of CuO NPs

The results of the antimicrobial assay demonstrate that CuO NPs exhibited the ability to inhibit the growth of all the bacterial strains tested as compared to the *C. unshiu* fruit peel extract and the precursor salt. The minimum inhibitory concentration (MIC) values of the synthesized NPs against *Bacillus. cereus, Staphylococcus. aureus, Escherichia . coli,* and *Salmonella. typhimurium* are displayed in **Table** **1**. The biosynthesized CuO NPs exhibited moderate activity against the gram‐positive strains *B. cereus* (MIC = 15.62 µg mL^−1^) and *S. aureus* (MIC = 62.50 µg mL^−1^), but it showed significant activity against *E. coli* (MIC = 7.81 µg mL^−1^). In contrast, it showed moderate activity against the gram‐negative strain *S. typhimurium* (MIC = 31.35 µg mL^−1^). Moreover, the extract exhibited moderate antimicrobial activity against *B. cereus* and *S. typhimurium (*MIC* = *62.25 µg mL^−1^) but not against *E. coli* and *S. aureus.* This suggests that *C. unshiu* fruit peels exhibit antimicrobial activity; however, it differs with bacterial strains. These results show that with low MICs for *E.* *coli*, it revealed their high susceptibility to CuO NPs, but higher MIC values for S. *aureus* and *S. typhimurium* confirmed their resistance; however, they show enhanced activity compared to the extract. These results align with those of Padil and Cernik. (2013),^[^ ^18^ ^]^ who indicated that CuO NPs synthesized from gum karaya showed high activity against *E. coli* with an MIC of 103 ± 4.7 µg mL^−1^.

**Table 1:** Antimicrobial and cytotoxicity activities of the synthesized CuO NPs. The bold values indicate significant antimicrobial activity (MIC < 125 µg mL^−1^).

	MIC ( µg mL^−1^)				IC_50_ values ( µg mL^−1^ ± SD)	
	*B. cereus*	*S. aureus*	*E. coli*	*S. typhimurium*	HEK293 cells	A549cells
*C. unshiu* extract	**62.25**	125	125	**62.50**	Not tested	Not tested
CuO NPs	**15.62**	62.50	**7.81**	**31.25**	34.90 ± 0.73	25.48 ± 0.28
Cu(NO_3_)_2_.3 H_2_O	250	62.50	**31.25**	250	10.38 ± 0.80	12.24 ± 0.72
Ciprofloxacin	<0.02	<0.02	<0.02	<0.02	Not tested	Not tested
Doxorubicin	Not tested	Not tested	Not tested	Not tested	1.54 ± 0.07	1.02 ± 0.02

Using *Tabarnaemontana divaricate* leaf extract with a zone diameter of 17 ± 1 mm at a concentration of 25 µg mL^−1^, similar results were obtained, showing that the highest zone of inhibition was found in *E. coli* for synthesized CuO NPs.^[^ ^19^ ^]^ These results show that CuO NPs were not only significant towards gram‐positive strains as demonstrated by Kaningini et al.,^[^ ^20^ ^]^, but they can also be significant toward gram‐negative strains. The efficacy of the activity of CuO NPs on bacterial strains was found to be dependent on the size of the NPs,^[^ ^21^ ^]^ the incubation time, and the thickness of the cell wall.^[^ ^22^ ^]^ The antimicrobial activity of CuO NPs obtained from *Gloriosa superba* leaf extract was investigated against *E. coli*, *S. aureus*, *Klebsiella. aerogenes,* and *Pseudomonas. desmolyticum*. Significant activity was observed against all the bacterial strains; however, there was no conclusive study to propose a mechanism of action .^[^ ^23^ ^]^

The cytotoxic effects of the NPs on HEK293 and A549 cells were evaluated using the MTT assay. The results showed that the synthesized CuO NPs had no effect either on human or cancerous cells. The IC50 of HEK293 cell lines was 34.90 ± 0.73 µg mL^−1^, and for A549 cell lines, it was 25.48 ± 0.28 µg mL^−1^, as shown in Table 1. In comparison, doxorubicin, an anticancer drug, had IC50 values of 1.54 ± 0.07 μg mL^−1^ for HEK293 cell lines and 1.02 ± 0.02 μg mL^−1^ for A549 cell lines, indicating greater cytotoxicity in all cell types compared to the fabricated NPs. Additionally, compared to synthesized NPs, Cu(NO_3_)_3_.3 H_2_O was more toxic to both cell lines. These results suggest the potential of plant‐based CuO NPs as chemotherapy drugs; however, there is a need for further assessment for effectiveness and safety prior to clinical application.

### 2.2 CuO NPs Characterization

**Figure 1:**
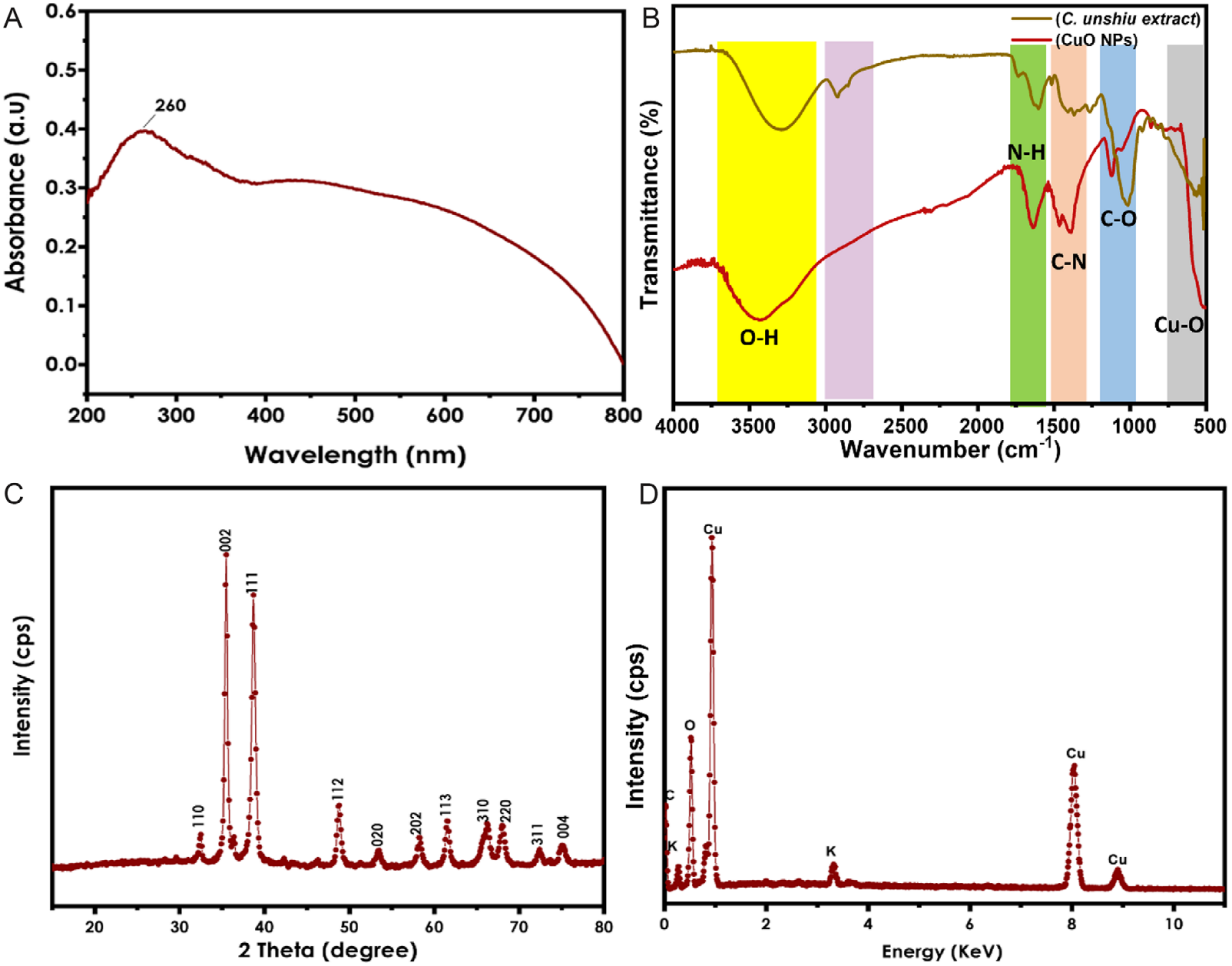
Characterization of the synthesized NPs A) UV–vis, B) FTIR, C) XRD, and D) EDS.

The UV–vis spectrum of the biosynthesized CuO NPs using *C. unshiu* fruit peel extract is shown in **Figure** **1**A The colloidal suspension after reduction exhibited a strong absorption band in the range of 200–400 nm, with an absorption feature centered at 260 nm and a weak absorption band around 450 nm. The 260 nm represents the n → π* transition of the carbonyl group of the extract's stabilizer, while the weak broad band from 400–700 nm indicates the formation of CuO NPs. These peaks confirm the production of carbonyl group‐stabilized CuO NPs. These results align with studies suggesting the formation of CuO NPs between 200 and 350 nm.^[^ ^24^ ^]^ The peaks correspond to the intrinsic band‐to‐band transitions in CuO NPs, confirming their successful synthesis.^[^ ^25^ ^]^

**Figure 2:**
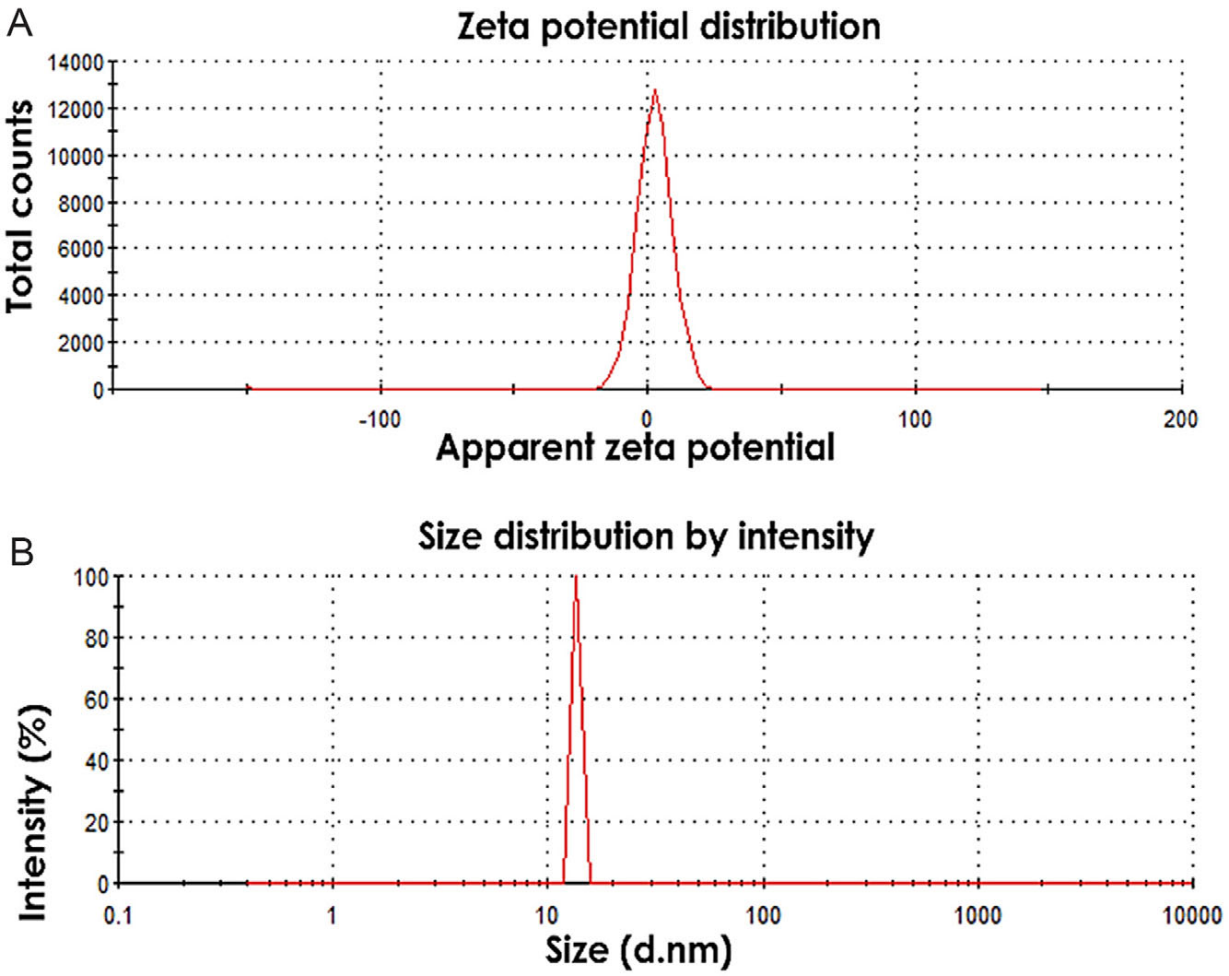
A) Zeta potential and B) hydrodynamic size of the prepared CuO NPs.

**Figure** **2**B represents the Fourier transform infrared (FTIR) of the fabricated NPs and the extract, which depicts the functional groups present in the *C. unshiu* fruit peel and their interaction with Cu ions during the CuO NPs fabrication. The hydroxyl group peak shifted from 3370 to 3491 cm^−1^ on the *C. unshiu* extract and the fabricated NPs, respectively. The shifting suggests the involvement of the hydroxyl groups from phenolic compounds in reducing and stabilizing copper ions. The peak at 2942cm^−^^1^, which represents the stretching of C—H, O—H stretching, and C=O stretching, disappeared in the CuO NPs wherein the disappearance might be due to the fact that the phytochemicals thereof are responsible for reducing the copper ions.^[^ ^26^ ^]^ The shifting (N—H), intensifying (C—N), and shortening (C—O) of the peaks from *C. unshiu* extract to CuO NPs indicate the involvement of *C. unshiu* fruit peel extract phytochemicals as reducing, capping, and stabilizing agents during green synthesis.^[^ ^27^ ^]^ The CuO stretching band confirming the synthesis of CuO NPs was observed at 535 cm^−^^1^. The presence of these functional groups underscores the extract's dual function as both a reducing and capping agent.

Figure 1C displays the X‐ray diffraction (XRD) pattern of CuO NPs which were synthesized by using copper (II) nitrate trihydrate and *C. unshiu* fruit peel extract as a reducing, capping, and stabilizing agent. The XRD spectrum was recorded within the 2*θ* range of 20° to 80°. The presented XRD pattern is associated with the monoclinic structure of CuO NPs with planes confirmed using JCPDS No. 01‐086‐8837.^[^ ^28^ ^,^ ^29^ ^]^ Based on the diffraction patterns, the concentric rings corresponding to reflections at (110), (002), (111), (112), (020), (202), (113), (310), (220), (311), and (004) confirmed the crystallinity of the as‐synthesized CuO NPs. The results are concurrent with previously reported works.^[^ ^19^ ^,^ ^30^ ^]^ The crystallite size of the monoclinic crystal was determined using the Debye–Scherer equation. All XRD diffraction peaks that are depicted in Figure 1C are related to CuO NPs without any impurity peak, which confirms the purity of the NPs. The estimated average crystallite size was 21.10 nm.

The results of the energy dispersive X‐ray analysis (EDX) depicted in Figure 1D outline the presence of four elements within the synthesized CuO NPs powder. Notably, the analysis revealed peaks for copper Cu (65.44%) and oxygen O (19.18%), substantiating the preparation of CuO NPs utilizing the *C. unshiu* peel extract. Consistent results were also reported by Kaningini et al.,^[^ ^31^ ^]^ further supporting the reliability of the CuO NP synthesis. Furthermore, the evident peaks for carbon (C) at 12.11% and potassium (K) at 3.27% are ascribed to the presence of bioactive compounds from the *C. unshiu* peel extract on the surface of the synthesized CuO NPs. This observation validates the influence of phytochemicals from the *C. unshiu* peel extract in the reduction and stabilization of the synthesized CuO NPs.

The hydrodynamic size, polydispersity index (PDI), and zeta potential of the synthesized CuO NPs were determined using dynamic light scattering (DLS) analysis, and the results are shown in Figure 2A–B. The zeta potential of the biosynthesized CuO NPs exhibited a sharp peak at +10.45 mV, indicating a positive surface charge and dispersion in the medium (deionized water) (Figure 2A). The size distribution of the synthesized CuO NPs ranged from 12 to 35 nm, as depicted in Figure 2B. The average particle size was determined to be 32.5 nm, slightly larger than the size obtained using XRD.^[^ ^32^ ^]^ This is attributed to the inclusion of biomaterials covering the surface of the CuO NPs in the measured size, along with the influence of particle‐dispersant molecule interactions. The positive charge might suggest that the pH of the dispersion medium (deionized water) was lower than the isoelectric point of CuO NPs, which resulted in protonation of CuO.

**Figure 3:**
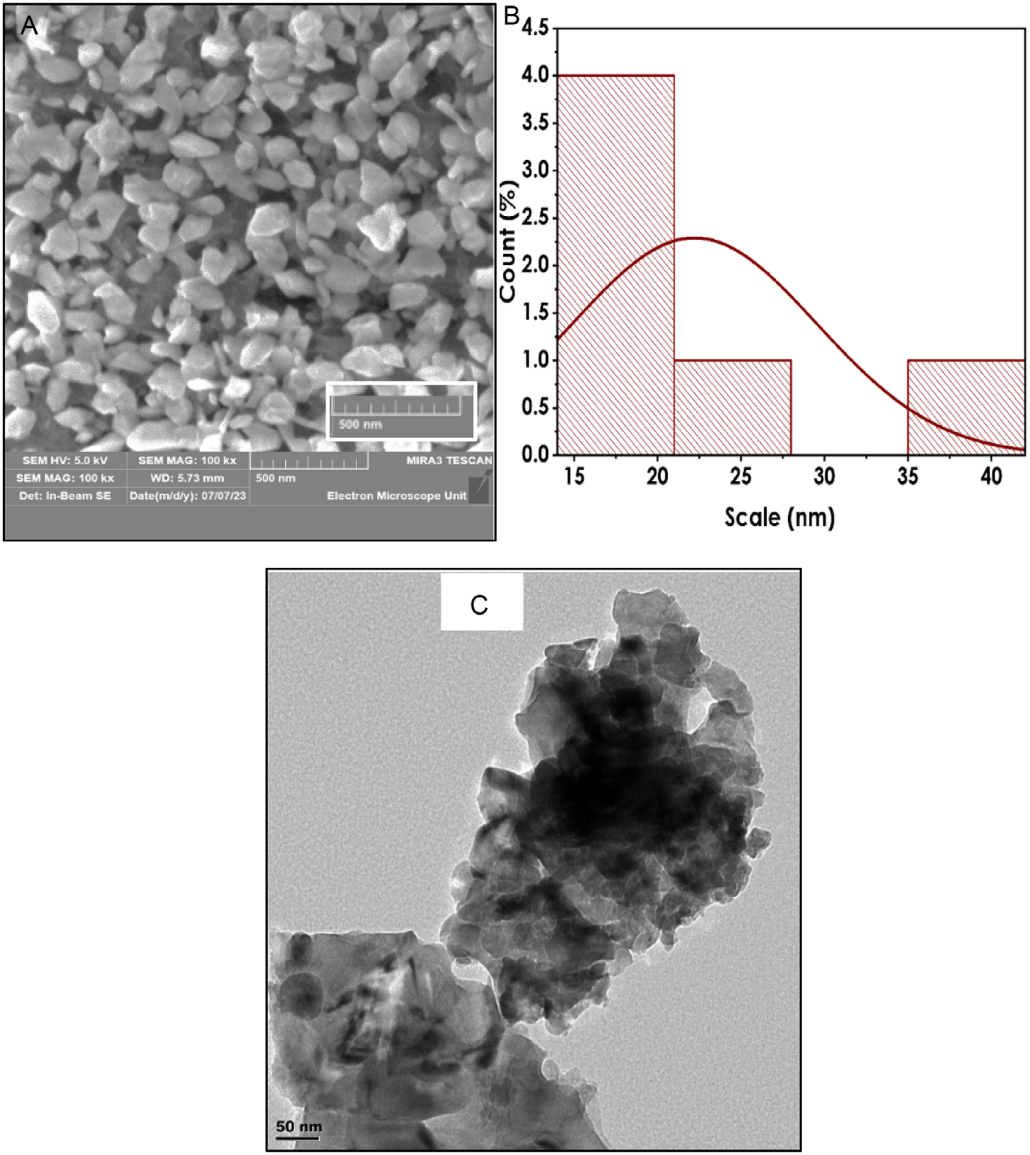
Morphology characterization: A) SEM micrograph, B) Size distribution, and C) TEM micrograph.

The polydispersity was found to be 0.586, confirming the good quality of the synthesized CuO NPs in terms of a well‐defined dimension with moderate monodispersity. However, monodispersity has also been attributed to aggregation or agglomeration during nanoparticle synthesis.^[^ ^20^ ^]^ The shape, size, and morphology of the biosynthesized CuO NPs were examined using scanning electron microscopy (SEM) and transmission electron microscopy (TEM), as shown in **Figure** **3**A–C.

The SEM image (Figure 3A) of the biosynthesized CuO NPs revealed the presence of variously sized, well‐dispersed quasispherical NPs. Analysis using ImageJ software indicated that the sizes of the NPs ranged from 15 to 40 nm, with an average diameter of 23 nm, as depicted in Figure 3B. These findings are consistent with the results obtained from XRD and DLS. The TEM results (Figure 3C) showed that the small particles were aggregated, possibly due to Oswald ripening, a process in which adjacent small NPs spontaneously coalesce into larger particles through heterogeneous nucleation and further metal ion reduction.^[^ ^33^ ^]^

### 2.3 UHPLC‐QTOF‐MS Analysis

Green synthesis of different CuO NPs from different plant extracts has been reported for different applications.^[^ 34, 35, 36 ^]^ The fundamental principle behind green synthesis is the ability of phytochemicals present in the plant extracts to reduce metal ions and stabilize them into NPs.^[^ ^4^ ^,^ ^37^ ^]^ However, the mechanism underlying this green synthesis remains poorly understood. Therefore, comprehending the phytochemical profile is crucial for elucidating the roles these compounds play in the green synthesis of CuO NPs, potentially enhancing the efficiency and effectiveness of nanoparticle production.

**Figure 4:**
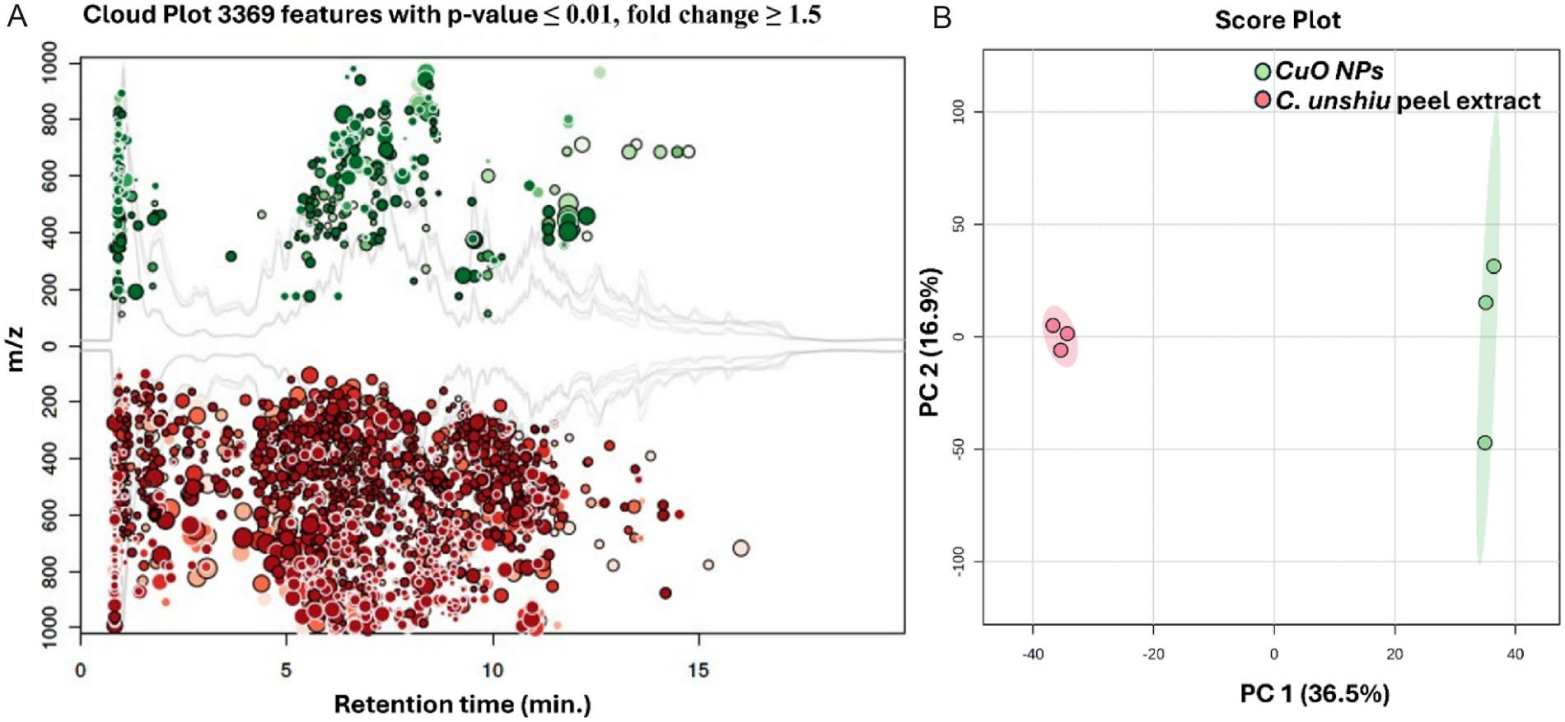
A) shows a cloud plot comparing metabolite compositions between the extract before and after green synthesis, with a significant *p*‐value of < 0.01 and a fold change exceeding 1.5. B) PCA scores scatterplot illustrating PCA 1 and PCA 2, explaining 53.4% of the variation. The control sample exhibited a scattered pattern (in red), whereas the treated sample displayed a clustered pattern (in green).

This study employed UHPLC‐QTOF‐MS and a molecular networking approach to analyze the phytochemical composition of *C. unshiu*. This methodology enabled comprehensive profiling of *C. unshiu*'s chemical constituents, both before and after green synthesis, utilizing UHPLC‐QTOF‐MS profiling, chemometric analysis, and molecular networking on a large scale. The differences between the composition of the crude extract and the supernatant after synthesis were examined using chemometric models. Principal component analysis (PCA) facilitated data dimensional reduction, enabling the visual interpretation and further exploration of the multidimensional data in a 2D representation. The PCA results revealed distinct differences in metabolic profiles before and after synthesis, as shown in **Figure** **4**. This is evidenced by the clustering of the extract samples and the scattering of the supernatant samples in different regions of the axis, indicating a significant biological difference. The observed metabolic trends or differences could be attributed to the reaction between the plant extract and the salt precursor, resulting in the formation of CuO NPs. Additionally, to validate the PCA results, a cloud plot was computed to highlight the metabolic differences between the extract and supernatant after green synthesis. The cloud plot provided a clear visual representation of the metabolic variations and systematic trends among sample groups, showing a reduction of metabolites in the extract following green synthesis.

UPLC‐QTOF‐MS data was utilized to create and display molecular networks in cytoscape in order to investigate the chemical diversity composition of the *C. unshiu* plant extract. Molecular networking, a computational tool, classifies chemicals based on mass spectral similarities by grouping structurally related metabolites through their MS/MS fragmentation patterns. In the molecular network generated in this study, nodes represent consensus MS/MS spectra labeled with neutral precursor mass, and edges connect nodes with common fragmentation patterns, indicating structural similarity. The MS/MS spectra obtained from the *C. unshiu* extract were organized into a total of 653 nodes in the molecular network. Out of these, 338 nodes formed 38 distinct molecular families, with each family consisting of at least two interconnected nodes. The remaining 227 nodes did not cluster into molecular families and were represented as individual singlets at the bottom of the network, suggesting that they either have unique structures or their MS/MS spectra did not match any other compounds in the dataset. The formation of distinct molecular families in the network indicates the presence of structurally related metabolites in the *C. unshiu* extract. These families likely represent specific compound classes, such as flavonoids, terpenes, or alkaloids, based on their shared fragmentation patterns. The interconnected nodes within each family suggest that these compounds have similar structural features, which may offer significant details about chemical diversity and potential biological activities of the plant extract. Furthermore, the presence of singlets in the network highlights the presence of unique compounds in the extract that are not structurally related to the majority of the identified metabolites. These singlets may represent rare or novel compounds that warrant further investigation for their potential biological significance.

**Figure 5:**
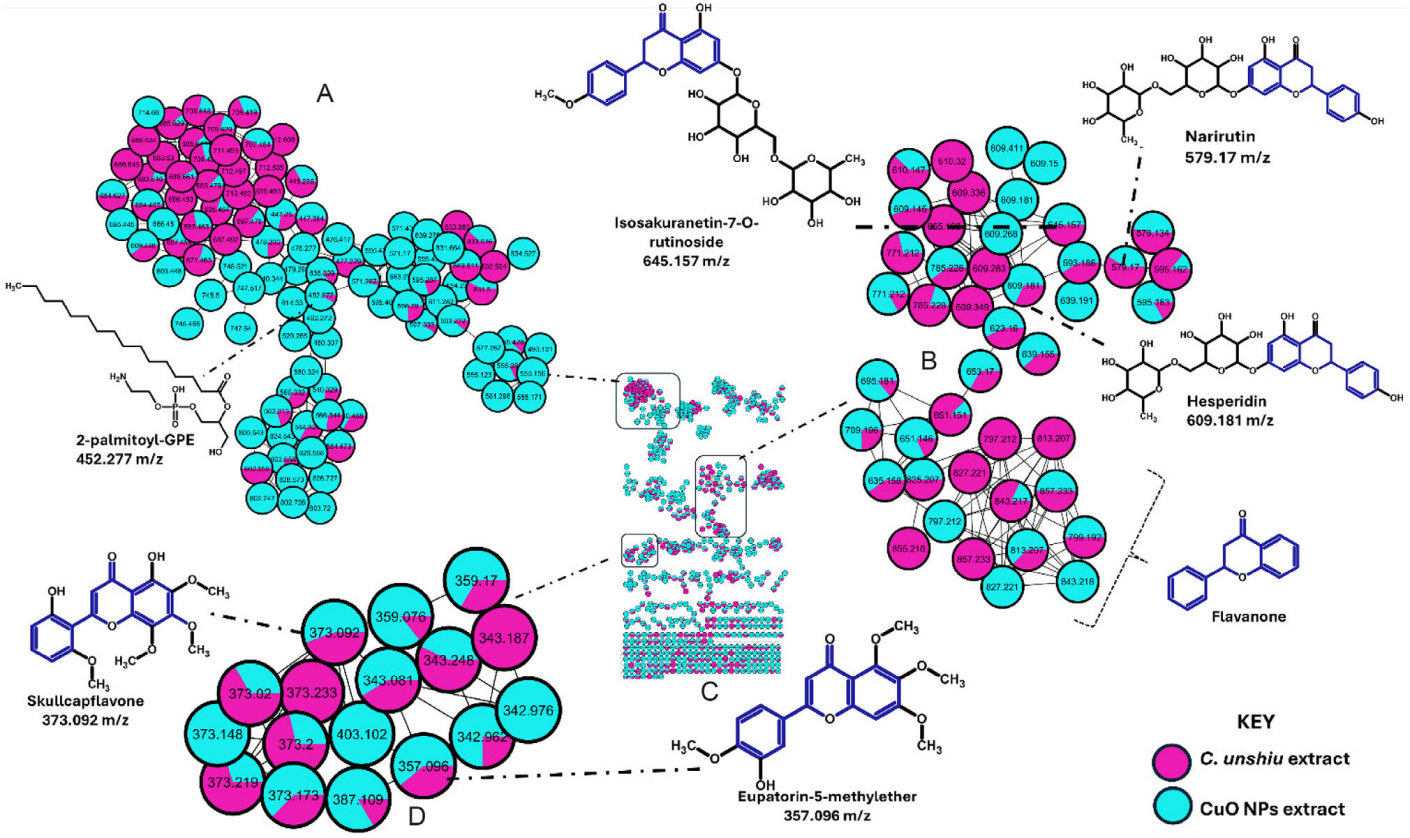
Molecular network of *C. unshiu* fruit peel extracts before and after nanoparticle synthesis, analyzed by UHPLC‐QTOF‐MS/MS using negative electrospray ionization mode. Node colors indicate the respective plant extracts studied, while the pie chart within each node illustrates the relative abundance of a given metabolite across the plants.

*C. unshiu* is known for its diverse range of chemicals, including flavonoids, carotenoids, essential oils, vitamins, minerals, and organic acids, which have both nutritional and medicinal value. This study's molecular network created a visual representation of the different compound families found in the plant extract (see **Figure** **5**). The *C. unshiu* extract was particularly abundant in flavonoids. Among the flavonoids identified in this study were isosakuranetin‐7‐O‐rutinoside (*m/z* 645.15 [M‐H]^−^), hesperidin (*m/z* 609.18 [M‐H]^−^), skullcapflavone II (*m* */* *z* 373.09 [M‐H]^−^), homoorientin (*m/z* 447.09 [M‐H]^−^), eupatorin‐5‐methylether (*m/z* 357.09 [M‐H]^−^), scoparin (*m/z* 461.10 [M‐H]^−^), and vitexin (*m/z* 431.09 [M‐H]^−^). These compounds are recognized for their antimicrobial activity properties, which are crucial for reducing metal ions during nanoparticle synthesis.

The presence of *ortho*‐substituted hydroxyl groups in these flavonoids augments their redox capabilities and facilitates the generation of robust NPs. Previous studies have shown that flavonoids play a significant role in determining the size and structure of synthesized NPs, resulting in well‐dispersed formations suitable for diverse applications, including antimicrobial and drug delivery systems. Furthermore, several spectral nodes within these clusters, specifically *m/z* 123.3, 785.228, 771.212, 653.17, 797.212, 825.207, and 651.146, eluded annotation using the Global Natural Product Social Molecular Networking (GNPS) spectral library. Nevertheless, their structural proximity to known flavonoids such as narirutin (*m/z* 597) and hesperidin (*m/z* 609) suggests a potential association with glycosylated flavonoids. The existence of these unclassified nodes underscores the chemical intricacy and diversity of *C. unshiu*, likely harboring a multitude of unexplored or under‐researched secondary metabolites.

## 3 Conclusion

The study illustrates the successful utilization of *C. unshiu* fruit peel extract for green, biocompatible, and low‐cost synthesis of CuO NPs. Characterization through UV–vis and DLS confirmed the stability of the synthesized NPs. Elemental analysis via EDX and structural assessment through XRD validated the formation of monoclinic CuO NPs. Moreover, the synthesized NPs exhibited significant antimicrobial activity against various pathogens, including *B. cereus, S. aureus, E. coli, and S. typhimurium*, with *E. coli* being particularly susceptible. The application of molecular networking in profiling metabolites from the extract has proven invaluable for identifying various compounds through spectral library matching. Additionally, a thorough examination of the molecular families of related molecules facilitated the putative identification of compounds with potential for unique nanoparticle synthesis. This approach underscores the significant promise of molecular networking in the dereplication of untargeted metabolomics data, paving the way for the discovery of novel compounds with diverse applications. Moreover, the findings suggest that CuO NPs could serve as a viable alternative to traditional antibiotics in combating bacterial infections, particularly in public health and agricultural settings where antibiotic resistance is a growing concern. Their efficacy against multidrug‐resistant strains underscores their potential role in sustainable agriculture and public health. Furthermore, understanding the mechanisms of action could lead to the development of improved formulations that enhance antimicrobial effectiveness while minimizing the risk of resistance development. This research contributes to the broader field of nanobiotechnology, highlighting the importance of plant‐derived materials in synthesizing NPs with significant biological activity.

## 4 Experimental Section

4.1

4.1.1

##### Microbial Strains, Cells, and Reagents

The materials and strains were as listed by Mafhala et al.^[^ ^25^ ^]^. *S. aureus* (ATCC: 25,923), *B. cereus* (ATCC: 10,876), *E. coli* (ATCC: 25,922), and *S. typhimurium* (ATCC: 39,183) bacterial strains were obtained from Microbiologics KWIK STIK (ANATECH, South Africa). The copper (II) nitrate trihydrate (Cu (NO_3_)_2_.3H_2_O) was purchased from Sigma‐Aldrich (Darmstadt, Germany). *C. unshiu* fruits were bought from the local supermarket (Roodepoort, Johannesburg), and the peels were collected. NaOH was procured from Glassworld.

##### *C. Unshiu* Peel Extraction

The peel extraction was carried out exactly as stated by Mafhala et al.^[^ ^25^ ^]^ Briefly, the peels were dried at room temperature, then ground into powder. Subsequently, distilled water was used for extraction for better extraction of polyphenols. The extract was kept at 4 °C until use.

##### Fabrication of CuO NPs Using *C. Unshiu* Fruit Peel Extract

Produced with minor adjustments, the CuO nanoparticle was made in line with Kaningini et al.^[^ ^31^ ^]^. This involved the addition of 0.75 g of Cu(NO_3_)_2_.3H_2_O to 20 ml of the aqueous *C. unshiu* peel extract to ensure sufficient availability of phytochemicals for effective reduction and stabilization of the NPs. Following heating the solution at 60 °C, the solution was stirred at 200 rpm till the appearance of dark paste. The pH of the solution was maintained and kept at 7.5 using NaOH. After cooling the paste, it dried up for 2 h in an oven at 60 °C. Afterward, the fabricated NPs were heated for 2 h at 300 °C for the purpose of stabilizing and removing excess water and other unwanted matter. All the mentioned conditions provided optimal conditions for consistent NP formation without excessive dilution. The acquired yield of 246.6 mg of CuO NPs was then stored in airtight Eppendorf tubes in a cool dry place for further analysis.

##### CuO NPs Characterization

The following characterization techniques were used as outlined by Mafhala et al.:^[^ ^25^ ^]^ FTIR spectroscopy and DLS technology using a Malvern Zetasizer Nano ZS (Malvern Instruments Ltd., GB). UV–vis spectroscopy (Shimadzu UV‐1800), a Rigaku SmartLab X‐ray diffractometer, an EDX (Oxford Instruments) coupled with a SEM using a Nova NanoSEM (Oxford Instruments, Oxfordshire, United Kingdom), high‐resolution transmission electron microscopy, a Tecnai F20 FEG‐TEM.

##### Assessment of the MIC

The method of broth microdilution as outlined by Mafhala et al.^[^ ^25^ ^]^ was utilized to evaluate the CuO NPs’ antimicrobial activity on four bacterial strains, namely *B. cereus*, *S. aureus*, *E. coli*, and *S. typhimurium*. In short, the same procedure was used with the replacement of AgNPs with CuO NPs. Wherein, a stock concentration of 2 mg mL^−^^1^ was achieved by dissolving 2 mg of CuO NPs in deionized water. For concentration variation, 100 µL of the nutrient broth was added to each of the 96 well plates and diluted accordingly. Subsequently, 100 μL of the standard inoculum at McFarland No. 1 of bacteria was added. The CuO NP concentration ranged from 3.90 to 500 µg mL^−1^. After allowing the bacteria to grow for 24 h at 37 °C, resazurin (20 μL of 0.2 mg m^L^ ^−1^) was added as an indicator of bacterial growth, and the plates were incubated for an additional hour. Viable bacterial growth converting resazurin to resorufin results in the formation of a pink formazan, whereas a dark‐blue color indicates bacterial suppression. The MIC was determined as the minimal antibacterial concentration that preserved a blue hue, signifying microbial suppression. The antibacterial properties of Cu(NO_3_)_2_·3H_2_O, the extract, and ciprofloxacin were also examined for comparison.

##### Evaluation of the Cytotoxicity for CuO NPs

The MTT assay was used to evaluate the cytotoxic potential of the extract, doxorubicin, and CuO NPs, Cu (NO_3_)_2_.3H_2_O, following a method by Mafhala et al.^[^ ^25^ ^]^ The same cell lines (lung cancer cells (A549) and human embryonic cells (HEK 293)) were used as in Mafhala et al.^[^ ^25^ ^]^ Briefly, subsequent to cell incubation in 96‐well microplates, CuO NPs were applied in the range of 10–100 µg mL^−1^ to the cells. Each well received 20 μL of MTT solution made with 5 mg mL^−1^ PBS as well as doxorubicin and the extract, which acted as the positive and negative control respectively. Following an additional 4 h of microplate incubation, 100 µL of DMSO was added to the mixture to dissolve the formazan crystals, and the mixture was left for 1 h. Notably, before use, 10% FBS and 1% penicillin‐streptomycin solution were added to sterile Dulbecco's Minimal Essential Medium (DMEM, Gibco) for the cultivation of A549 and HEK 293 cells. Once incubated, plates were read by an enzyme‐linked immunosorbent assaymachine (VarioskanFlash, ThermoFisher Scientific, Vantaa, Finland), and the percentage of viable cells was determined.

##### UHPLC‐QTOF‐MS Analysis

For LC–MS analysis, samples were heated (as part of the green synthesis procedure for CuO NPs), centrifuged, and supernatant separated from the precipitate. The supernatant was later diluted, further centrifuged (4000 rpm, 15 min) and transferred into LC–MS vials for analysis. Additionally, *C. unshiu* peel extract was also diluted to obtain comparable results. A LC‐QTOF tandem MS instrument (LCMS‐9030 QTOF, Shimadzu Corporation, Kyoto, Japan) fitted with a Shim‐pack Velox C18 column (100 mm × 2.1 mm, particle size 2.7 µm) was utilized for the analysis of the prepared extracts. A column oven set to 55 °C for 30 min was used to separate the analytes.^[^ ^38^ ^]^ The injection volume was set to 3 μL. Component separation in the plant extract was achieved using binary gradient elution, with mobile phase A consisting of 0.1% (v/v) formic acid in ultrahigh purity water and mobile phase B of 0.1% formic acid in acetonitrile with a flowrate of 0.3 mL min^−1^. In particular, the gradient elution was as follows: Eluent B was maintained at 5% from 0 to 3 min, increased linearly from 5% to 40% between 3 and 8 min, further increased to 95% between 8 and 23 min, held isocratically at 95% from 23 to 25 min, returned to 5% from 25 to 27 min, and reequilibrated at 5% from 27 to 30 min. The eluents from chromatography were subjected to analysis using QTOF high‐definition MS operated in negative mode, with constant settings: 1.8 kV detector voltage, 4.0 kV interface voltage, 400 °C heat block, 300 °C interface, 3 L min^−1^ nebulization and dry gas flow, 280 °C DL temperature, and 42 °C flight tube temperature. Sodium iodide (NaI) was used as a calibration solution to ensure high mass accuracy. MS^1^ and MS^2^ spectra were acquired to cover all *m/z* values within the 100–1000 range, corresponding to the precursor *m/z* isolation window.

##### Classical Molecular Networking of *C. Unshiu* Fruit Peel Extract and CuO NPs

GNPS platform (http://gnps.ucsd.edu) was used to build molecular networks. The raw UHPLC‐QTOF‐MS/MS data were converted to mzML format prior to upload. MS^2^ fragment ions that were within ±17 Da of the precursor m/z were filtered out after submission. Only the four highest fragment ions within a ±50 Da range were selected for further analysis based on the MS/MS spectra. By establishing mass limitations of 0.02 Da for both MS^2^ fragment ions and precursor ions, the MS‐CLUSTER methodology facilitated data clustering. Edges representing the similarities between metabolites were established only when there were more than six matching peaks and a minimum cosine score of 0.7. With a maximum of 100 nodes per molecular family, nodes were kept in the network if they were among the top 10 most similar nodes to one another (TopK value of 10). Based on empirical calculations derived from precise mass and fragmentation patterns from the MS^2^ data, all matched and a few mismatched nodes were annotated. A review of the literature and searches in popular natural product dereplication databases, including KNApSAcK, were used to further validate these annotations. The Metabolomics Standards Initiative (MS^1^) level 2 requirements were adhered to in the annotation. Cytoscape 3.10.1 was used to visualize the networks that were produced.

## Conflict of Interest

The authors declare no conflict of interest.

## Author Contributions

**Livhuwani Mafhala**: methodology (equal); writing—original draft (lead), **Shohreh Azizi**: funding acquisition (lead); supervision: (equal); writing—review & editing (equal), **Garland More**: methodology (equal), **Ilunga Kamika**: writing—review & editing (equal); supervision (equal).

## Supporting information

Supplementary Material

## Data Availability

Data sharing is not applicable to this article as no new data were created or analyzed in this study.
