# Supplementary material for: Molecular Networking‐Driven Chemical Profiling, Characterization, and Antibacterial Effects of Cuo Nanoparticles Synthesized from Citrus Unshiu Peel Extract
Source: ChemistryOpen. 2025 Sep 19;14(12):e202500374. doi: 10.1002/open.202500374 (PMC12680546; doi:10.1002/open.202500374)
Supplement: Supplementary file 1 — Supplementary Material [file OPEN-14-e202500374-s001.pdf]

Supplementary data

Molecular Networking-Driven Chemical Profiling, Characterization, and Antibacterial Effects of CuO Nanoparticles Synthesized from *Citrus unshiu* Peel Extract

Livhuwani Mafhala[1], Shohreh Azizi[1], Ilunga Kamika[2]

The document contains the fragmentation trees acquired from Sirius of some compounds outlined in the manuscript.

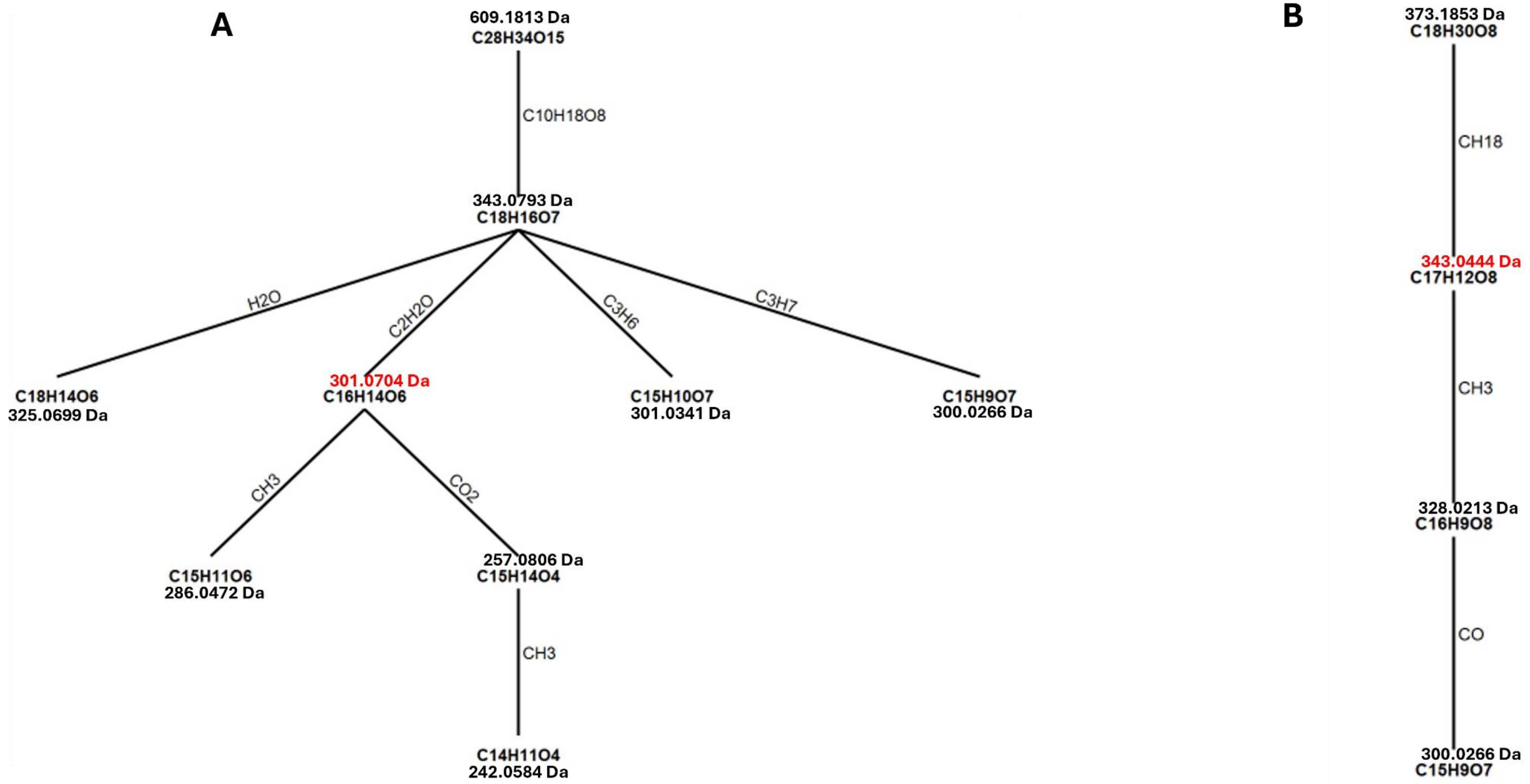
A
609.1813 Da
C28H34O15
C10H18O8
343.0793 Da
C18H16O7
H2O
C2H2O
C3H6
C3H7
C18H14O6
325.0699 Da
301.0704 Da
C16H14O6
C15H10O7
301.0341 Da
C15H9O7
300.0266 Da
CH3
CO2
C15H11O6
286.0472 Da
257.0806 Da
C15H14O4
CH3
C14H11O4
242.0584 Da
B
373.1853 Da
C18H30O8
CH18
343.0444 Da
C17H12O8
CH3
328.0213 Da
C16H9O8
CO
300.0266 Da
C15H9O7

c

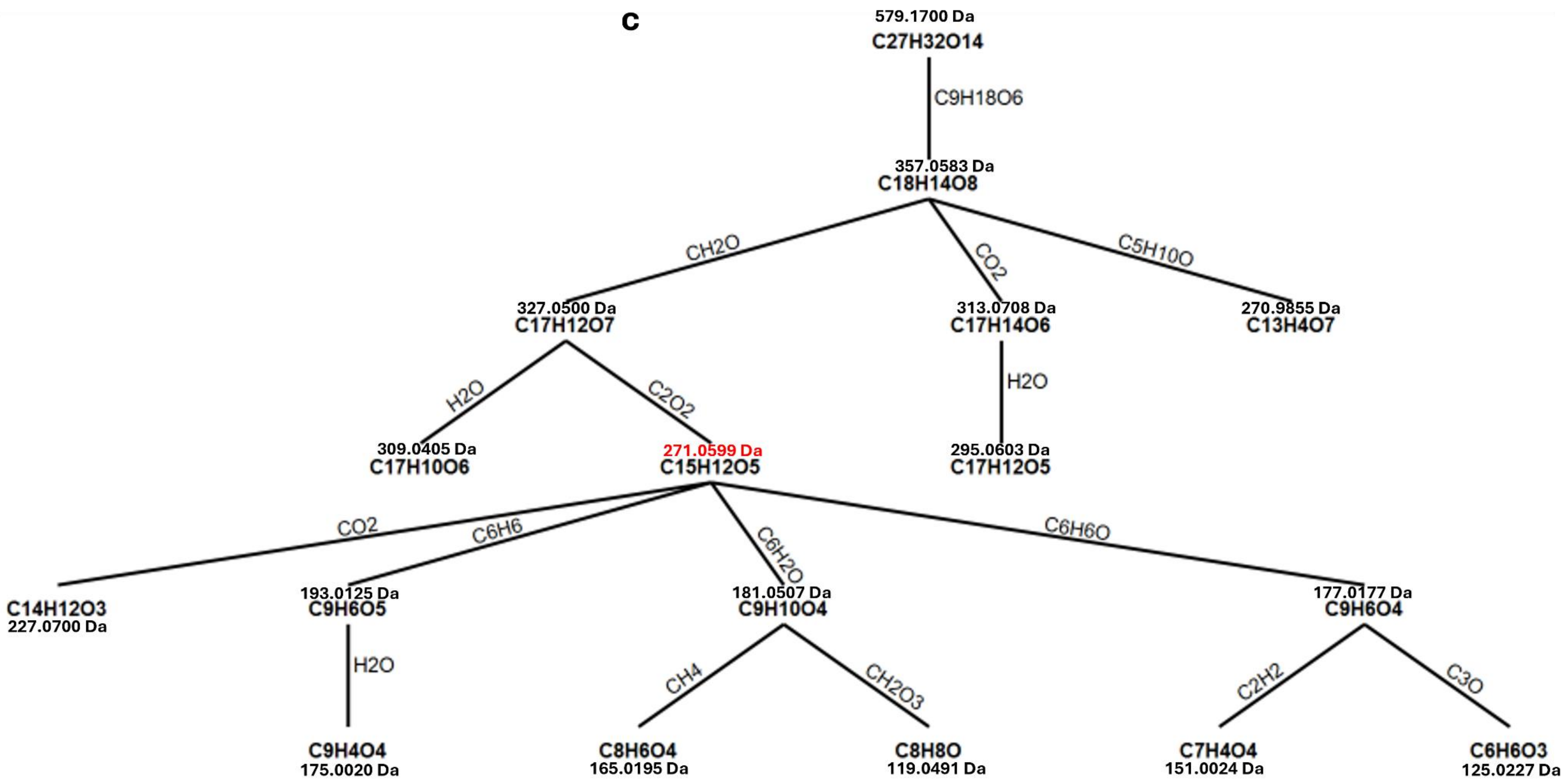

579.1700 Da
C27H32O14
C9H18O6
357.0583 Da
C18H14O8
CH2O
CO2
C5H10O
327.0500 Da
C17H12O7
313.0708 Da
C17H14O6
270.9855 Da
C13H4O7
H2O
C2O2
H2O
309.0405 Da
C17H10O6
271.0599 Da
C15H12O5
295.0603 Da
C17H12O5
CO2
C6H6
C6H2O
C6H6O
C14H12O3
227.0700 Da
193.0125 Da
C9H6O5
181.0507 Da
C9H10O4
177.0177 Da
C9H6O4
H2O
CH4
CH2O3
C2H2
C3O
C9H4O4
175.0020 Da
C8H6O4
165.0195 Da
C8H8O
119.0491 Da
C7H4O4
151.0024 Da
C6H6O3
125.0227 Da

D

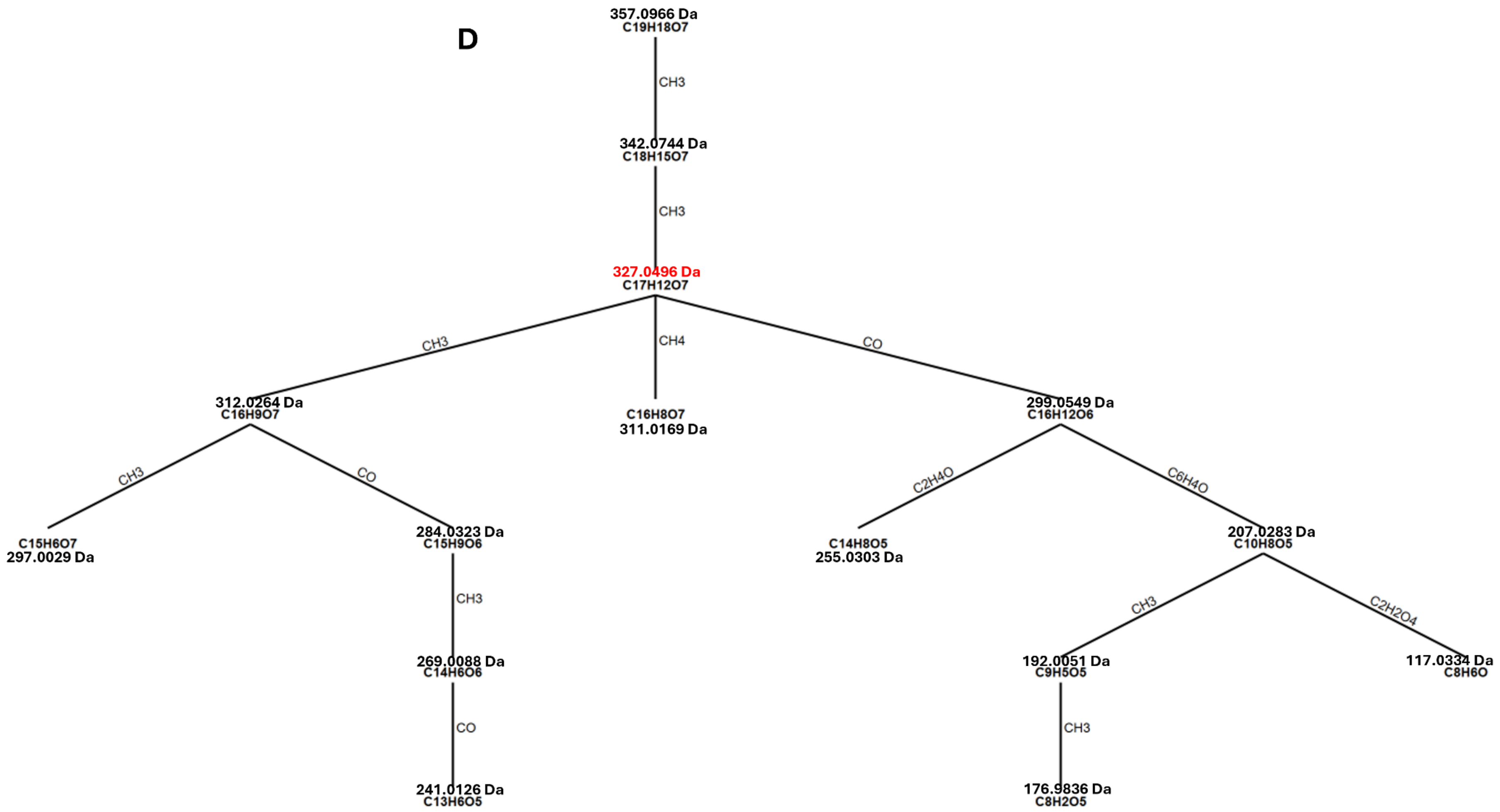
357.0966 Da
C19H18O7
CH3
342.0744 Da
C18H15O7
CH3
327.0496 Da
C17H12O7
CH3
CH4
CO
312.0264 Da
C16H9O7
C16H8O7
311.0169 Da
299.0549 Da
C16H12O6
CH3
CO
C2H4O
C6H4O
C15H6O7
297.0029 Da
284.0323 Da
C15H9O6
C14H8O5
255.0303 Da
207.0283 Da
C10H8O5
CH3
CH3
C2H2O4
269.0088 Da
C14H6O6
192.0051 Da
C9H5O5
117.0334 Da
C8H6O
CO
CH3
241.0126 Da
C13H6O5
176.9836 Da
C8H2O5

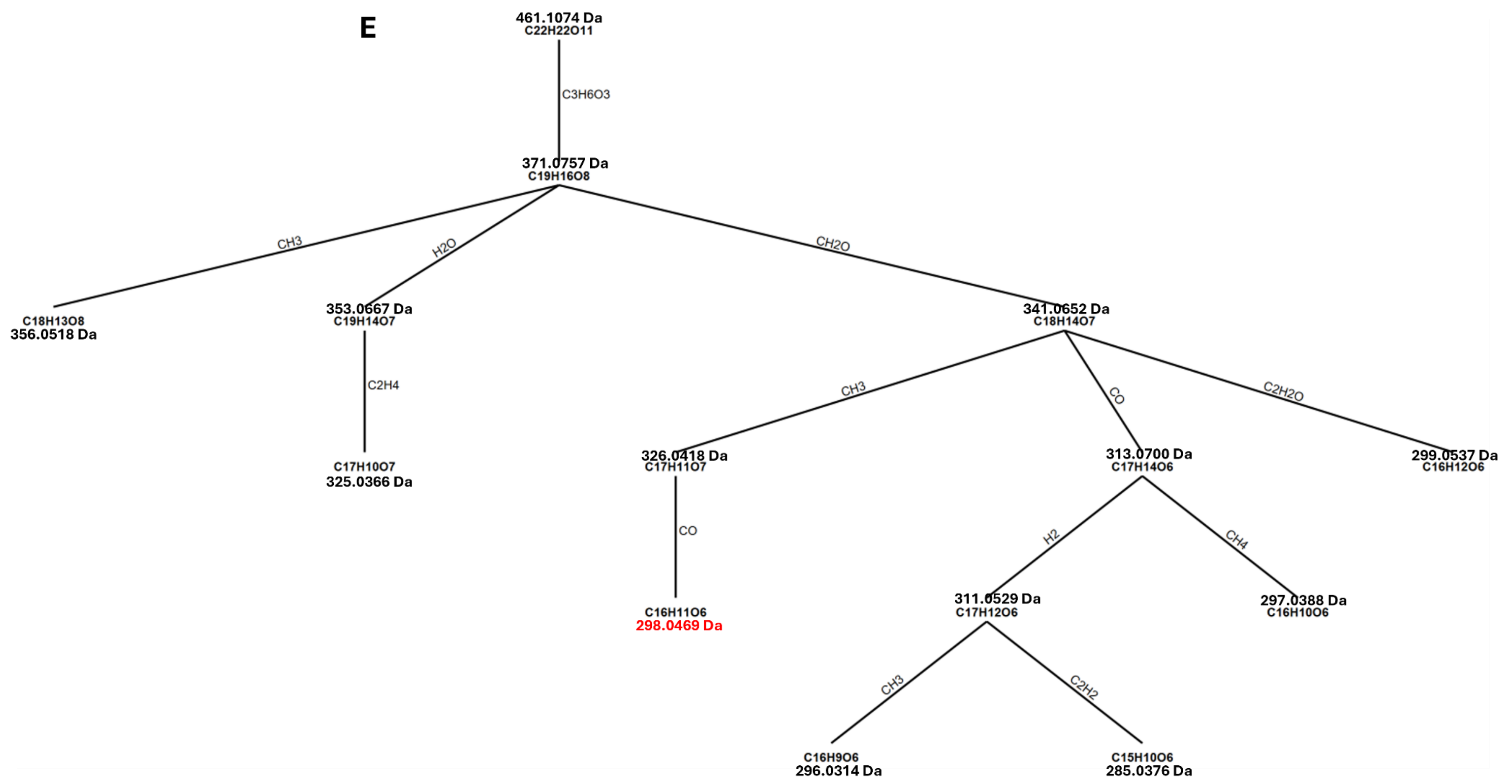
E
461.1074 Da
C22H22O11
C3H6O3
371.0757 Da
C19H16O8
CH3
H2O
CH2O
C18H13O8
356.0518 Da
353.0667 Da
C19H14O7
C2H4
C17H10O7
325.0366 Da
341.0652 Da
C18H14O7
CH3
CO
C2H2O
326.0418 Da
C17H11O7
CO
C16H11O6
298.0469 Da
313.0700 Da
C17H14O6
H2
CH4
311.0529 Da
C17H12O6
297.0388 Da
C16H10O6
299.0537 Da
C16H12O6
CH3
C2H2
C16H9O6
296.0314 Da
C15H10O6
285.0376 Da

F

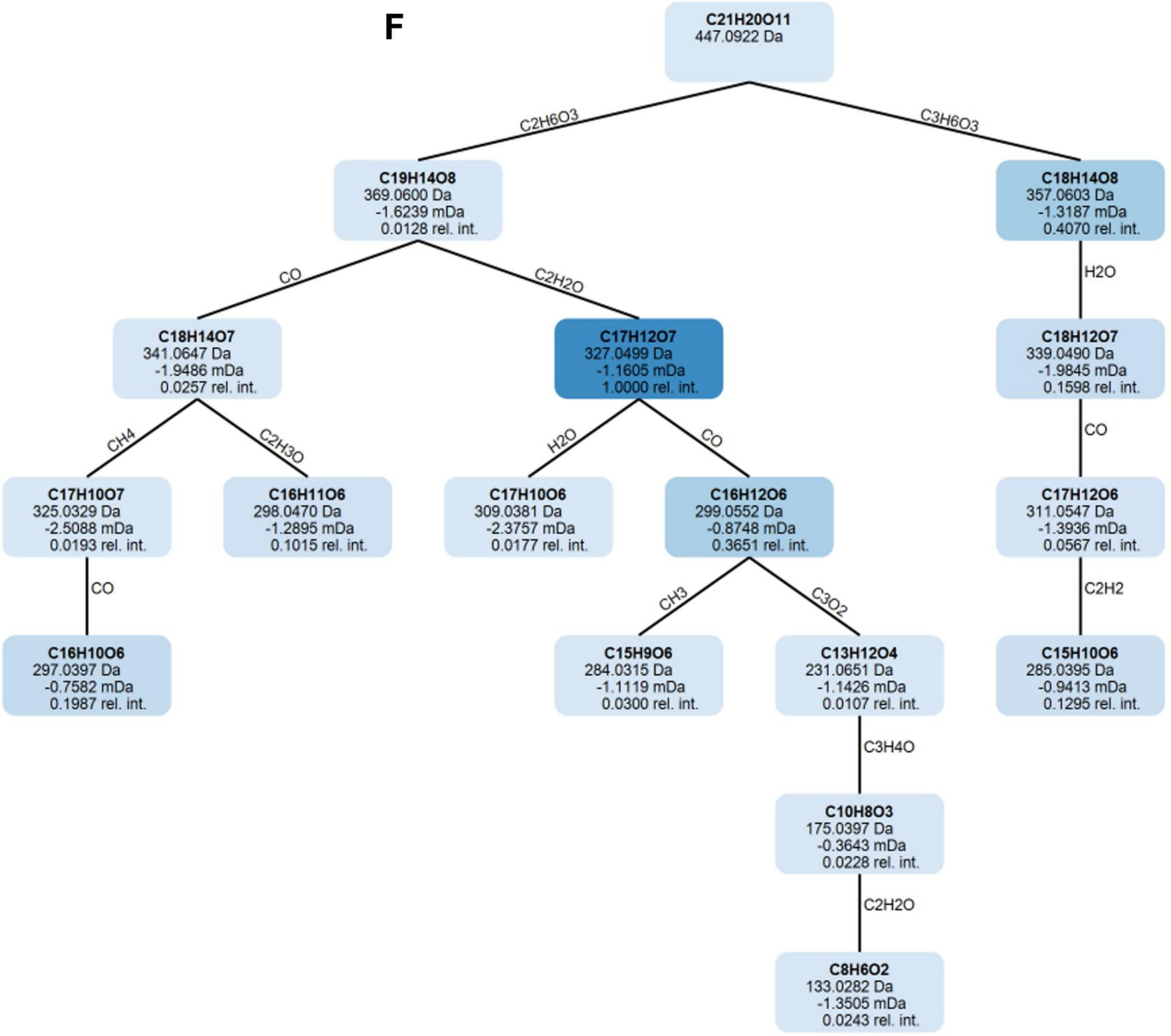
C21H20O11
447.0922 Da
C2H6O3
C3H6O3
C19H14O8
369.0600 Da
-1.6239 mDa
0.0128 rel. int.
C18H14O8
357.0603 Da
-1.3187 mDa
0.4070 rel. int.
CO
C2H2O
H2O
C18H14O7
341.0647 Da
-1.9486 mDa
0.0257 rel. int.
C17H12O7
327.0499 Da
-1.1605 mDa
1.0000 rel. int.
C18H12O7
339.0490 Da
-1.9845 mDa
0.1598 rel. int.
CH4
C2H3O
H2O
CO
CO
C17H10O7
325.0329 Da
-2.5088 mDa
0.0193 rel. int.
C16H11O6
298.0470 Da
-1.2895 mDa
0.1015 rel. int.
C17H10O6
309.0381 Da
-2.3757 mDa
0.0177 rel. int.
C16H12O6
299.0552 Da
-0.8748 mDa
0.3651 rel. int.
C17H12O6
311.0547 Da
-1.3936 mDa
0.0567 rel. int.
CO
CH3
C3O2
C2H2
C16H10O6
297.0397 Da
-0.7582 mDa
0.1987 rel. int.
C15H9O6
284.0315 Da
-1.1119 mDa
0.0300 rel. int.
C13H12O4
231.0651 Da
-1.1426 mDa
0.0107 rel. int.
C15H10O6
285.0395 Da
-0.9413 mDa
0.1295 rel. int.
C3H4O
C10H8O3
175.0397 Da
-0.3643 mDa
0.0228 rel. int.
C2H2O
C8H6O2
133.0282 Da
-1.3505 mDa
0.0243 rel. int.
